# Safe Hands 3.0: new tool to prevent healthcare associated infection

**DOI:** 10.1093/eurpub/ckac131.399

**Published:** 2022-10-25

**Authors:** C Valero-Ubierna, J Perez deRojas, E Marin Caba, D Martinez Bellón, S Gutierrez Linares

**Affiliations:** Medicine Preventive and Public Health, Universitary Clinic Hospital San Cecilio, Granada, Spain

## Abstract

**Background:**

Healthcare associated infection (HAI) is a Public Health problem. Hands of healthcare workers are a key for transmission. Compliance with WHO 5 moments for hand hygiene (HH) has been emphasized, but there are other aspects: appropriate use of gloves and healthy hands skin of professionals. Gloves protect the professionals but it may increase cross-transmission. Hands dermatitis is a handicap for the use of alcohol-based handrubs. Data collection and analysis, to have a good communication system to improve training, information and to get feedback from professionals are necessary. The aim of this work is to develop the Safe Hands 3.0 Project in a General Hospital.

**Methods:**

Preventive Medicine and Public Health Department carried out a descriptive study carried out in a 477-bed General Hospital in last semester 2021. Compliance with HH and the possible causes of non-compliance were assessed with observations and knowledge surveys included appropriate use of gloves in healthcare practice and the impact on hand in professionals. The surveys were carried out using Lime Survey for mobile devices and Google for WhatsApp.

**Results:**

From 550 observations of HH compliance for the WHO 5 moments is less than 40%. Half of non-compliance was due to inappropriate use of gloves. 266 surveys answered highlighting the need for continued training. Survey on hand skin health perception was answered by 182 professionals, 44% reported dermatological issues and 81% notice that their work damaged their hands skin. A corporate logo was created, use the hospital website and a structured Communication Plan for its implementation.

**Conclusions:**

To prevent HAI transmission, the concept of Hand Hygiene is limited. Safe Hands 3.0 Project comes up with a bundle of care: proper use of gloves, compliance the WHO 5 moments and skin care of healthcare workers’ hands. Update data management and a corporate communication plan with social networks is necessary to improve training and compliance.

**Key messages:**

• A bundle of care is the key for Safe Hands: proper use of gloves, compliance the WHO 5 moments and skin care of healthcare workers’ hands.

• Update data management and a corporate communication plan with social networks is necessary to improve training and compliance.

